# Water Content Variation Patterns of Fruits During Development and Their Effects on Fleshiness or Dryness for Fruits at Maturity

**DOI:** 10.3390/plants15142216

**Published:** 2026-07-21

**Authors:** Shunli Yu, Canran Liu, Hui Liu, Xiuli Gao, Chang’en Shang, Carol C. Baskin

**Affiliations:** 1 Key Laboratory of Vegetation and Environmental Changes, Institute of Botany, Chinese Academy of Sciences, Beijing 100093, China; gaoxiuli551070@163.com; 2 Arthur Rylah Institute for Environmental Research, Department of Energy, Environment and Climate Action, Heidelberg, VIC 3084, Australia; canran.liu@deeca.vic.gov.au; 3 South China Botanical Garden, Chinese Academy of Sciences, Guangzhou 510650, China; hui.liu@scbg.ac.cn; 4 University of Chinese Academy of Sciences, Beijing 100049, China; 5 College of Landscape Architecture, Central South University of Forestry and Technology, Changsha 410004, China; 18714596955@163.com; 6 Department of Biology, University of Kentucky, Lexington, KY 40506, USA; carol.baskin@uky.edu; 7 Department of Plant and Soil Sciences, University of Kentucky, Lexington, KY 40546, USA

**Keywords:** cuticle, epidermis, fleshiness, fruit, water content

## Abstract

The ecological origins of diverse fruit types remain unresolved due to lack of knowledge about variation patterns in fruit water content during fruit development and after fruit maturity. We hypothesized that distinctive water content variation patterns during fruit development mainly determine fleshiness or dryness of fruits at maturity. Controlled water evaporation experiment of fruits of 188 species (belonging to 144 genera and 72 families) was conducted to measure fruit drying days and water content. A phylogenetic tree was built for 29,760 seed plants of China using the R package V.PhyloMaker2 and phylogenetic generalized linear models and Pearson correlation analysis were used to explore the variation patterns of drying days of various fruit types and their mechanism. Water content in dry fruits decreases gradually during fruit development (i.e., prior to maturity, and before dehiscent fruits split), while water content in fleshy fruits (berries, at least the pericarp of drupes and rosehips) increases slightly or remains constant during fruit development. Significant correlations were found between fruit drying period and fruit height, surface area and fruit size. In general, the development of dryness for dry fruits at maturity is primarily attributed to declining fruit water content due to pericarp lignification and consequent fast water loss. Also, possible water imbalance between supply and loss especially for elongated or flaky dry fruits due to allometric growth between pedicel diameter (or fruit mass) and fruit surface area helps account for fruit dryness. Development of dry fruits may be induced by cold, dry and open habitats with strong light. Conversely, fleshiness of mature fleshy fruits is attributed to slow pericarp water loss rate due to low cuticle permeability and high fruit thickness, combined with stable high-water content maintained during fruit development. The evolution of fleshy fruits was induced by warm and humid habitats with weak or possibly strong light.

## 1. Introduction

Numerous studies have explored the biological characteristics of fleshy and dry fruits [1,2] and their ecological relationships with environmental factors [3,4,5]; however, the ecological origins of diverse fruit types and ecological factors surrounding their origin and early evolution remain unresolved due to lack of knowledge about variation patterns in fruit water content during fruit development and after fruit maturity. Therefore, much remains to be learned about the essential difference in water content dynamics during fruit development between these two fruit types, and the ecological causes underlying such differences, particularly in relation to water adaptability to heterogeneous habitats [4,5]. It is well established that dry fruits, though characterized by a low water content and dry pericarp at maturity, generally undergo a developmental phase with relatively higher water content and soft pericarp [6]. In contrast, fleshy fruits develop pericarps and accessory tissues that remain succulent and soft at maturity, still retaining high water content for extended periods. Yet, the temporal patterns of water content variation in the two fruit types both throughout development and after maturation, are still poorly understood. This limited knowledge hampers our ability to identify the long-term developmental and ecological mechanisms that determine whether fruits become fleshy or dry at maturity. Analysis of fruit water-retaining ability and water content dynamics between fruit types can advance our understanding on the origin and evolution of diverse fruit types and their responsible ecological factors [2,5]. Understanding the environmental and biological mechanism of fruit diversity is essential for the conservation of plant species and the sustainable use of plant resources.

Fleshy and dry fruits, two major plant functional groups with substantial phylogenetic differentiation, may have evolved through concerted convergence, with each type adapting to shaded and open habitats, respectively, in terms of water ecophysiology [7,8,9]. However, the strongest evidence and evolutionary drivers of this convergence still need to be identified [8]. The distribution of fleshy-fruited species and their close correlation with shaded habitats suggest that they or at least some of them may have originated in closed environments or forest understories [10,11], where solar radiation, air temperature, and airflow are relatively lower compared with open habitats. Thus, the origins of fleshy versus dry fruits are likely linked to differences in water and light availability shaped by environmental conditions [12]. Although soil moisture does not appear to significantly influence the distribution of fleshy-fruited species [10,13], temperature and rainfall do [5]. High water availability, together with increased photosynthate allocation, may have facilitated the evolution of fleshy fruits with larger seeds [12]. Nevertheless, the relative importance of abiotic factors such as light, temperature and wind, in determining fruit types, remains poorly understood. Likewise, the processes underlying the emergence of fruit fleshiness or dryness, and the mechanisms governing their development at ripening, are still only partially resolved.

Selection pressures that shape fruit traits, especially in fruit fleshiness or dryness, have puzzled ecologists and evolutionary biologists for decades [7,8,12,14,15]. Yet, these remain controversial topics due to limited knowledge of key characteristics such as fruit shape and pericarp traits, which directly influence water retention and transpiration [15]. Previous studies suggest that plant–animal interactions, correlated evolution among plant organs and environmental adaptations may provide important insights into the evolution of propagule traits in relation to fruit fleshiness or dryness [15,16]. However, the factors determining whether fruits mature as fleshy or dry, and the extent to which these outcomes are shaped by biotic factors such as internal structure and morphological properties, remain unclear [17]. We hypothesized that the development of fruit fleshiness results from a balance between water supply via the pedicel, water usage by tissue building and water loss through the pericarp [18]; however, the precise and common mechanisms remain unresolved. As for dry fruits, their water content dynamics and mechanisms during development are almost completely ignored [6,7,8,16]. Here, we hypothesized that the development of fruit dryness results from an imbalance between water supply via the pedicel and water loss through the pericarp due to allometric growth between pedicel diameter (or fruit mass) and fruit surface area [18]. Water transported from pedicels on twigs is ultimately partitioned among transpiration, photosynthesis and the construction of structural components. The barrier properties of pericarp cuticles are known to play a critical role in reducing water loss [19]. Nevertheless, whether allometric relationships or trade-offs exist between fruit water supply and loss, or whether fruit fleshiness is linked to other plant traits such as fruit shape and pericarp characteristics, are an open question [20].

The diversity of fruit shapes has long attracted scientific attention; for example, spherical forms are considered highly economical for packaging seeds [21]. Because a sphere minimizes surface area relative to volume, spherical fruits are also thought to promote water retention compared with other shapes. Thus, investigating fruit shapes in wild plants can provide valuable insights into water adaptation strategies of fruits in different environments. Notably, some of the earliest fossil angiosperm fruits were approximately globular [22]. This suggests that fruit morphology is not randomly formed but rather reflects a long-term adaptation and evolutionary responses to heterogenous environments [23].

In this study, we investigate the biological and environmental mechanisms underlying the development of fleshy and dry fruits. Specifically, we examine differences in fruit water content dynamics during development and after maturation, and test which biotic or abiotic factors shape these patterns. Because fruit shape is closely related to water loss, we also analyzed variation in fruit shape and its correlation with fruit types across wild seed plants in China. The overall aim of our study is to identify the ecological determinants of fruit fleshiness and dryness at maturity by integrating analyses of water content dynamics, fruit shape variation, and pericarp water flux measurement. We hypothesize that distinct patterns of water content variation, differences in fruit shape, and variation in pericarp permeability, mediated by adaptations to shaded versus open habitats in diverse climates [17], may drive the divergence between fleshy and dry fruits. More broadly, we seek to uncover the biological and environmental mechanisms of fruit fleshiness and dryness at maturity by exploring allometric growth relationships among plant organs and their water adaptation strategies across heterogeneous habitats.

## 2. Materials and Methods

### 2.1. Site Description

Fruit types and shapes of 29,760 seed plant species in China were analyzed (data attracted from Flora of China, 1994–2004). Water evaporation and water content measurement of fruits, involving 188 plant species (belonging to 144 genera and 72 families), was conducted from May 2022 to October 2024, mainly in Beijing Botanical Garden of the Chinese Academy of Sciences (42°00.550 N, 101°13.884 E, 50 m Alt.) [19], China. The water evaporation and water content measurement include a long-term fruit drying experiment touching 143 species and a short-term experiment (24 h, just for data of absolute water areal flux) touching 188 species (the above 143 species and another 45 species), of which 13 species were from other regions (5 from Guangdong Province, 4 Yunnan Province, 3 Hebei and 1 Xinjiang of China). The other fruits of 175 species were collected from the garden in Beijing.

### 2.2. Classification of Fruit Types and Shapes

Based on the water condition at fruit maturity, fruits were classified into two types: fleshy and dry. The former mainly included drupes, berries, pomes, pepos, aggregate fruits, hesperidia, rose hips and hesperidiums, and the latter mainly included capsules, achenes, samaras, pods, caryopses, nuts, silique, cremocarps, follicles, and utricles.

Based on the highest values of length-to-width ratio, length-to-height ratio and width-to-height ratio, combined with fruit diameter size, four fruit shape types were classified for 29,760 seed plant species: globular or spheroidal shapes (with all values of the three ratios < 2), elongated shapes (with one values of the three ratios > 2, and the height and width are almost equal), flaky shapes (with two values of the three ratios > 2, and values of length and width are much greater than the height) and small globular shapes (with diameter < 0.5cm).

### 2.3. Water Evaporation Experiment of Fruits—Fruit Water Content and Drying Days Measurement

At least three juvenile (individuals or groups, fruit number from 3 to 100) and three ripe fruits (same methods as the juvenile fruits) were collected randomly from the same individuals (or different individuals if fruits are too small) of each plant species by the method of numbering (individuals and fruits) at random within the restricted area. The fresh mass of fruits was measured immediately. Fruits were then allowed to dry in the open air in the laboratory under controlled experimental conditions with the same light (day: 750 ± 50 lx, night: 75 ± 10 lx), air flow speed (≤0.2 m/s), minimal average temperature variation (23 ± 3 °C), and similar daily air humidity (70 ± 10%), and they were weighed daily until they reached a constant mass (and the fruits of the 13 species from other places were measured using the same method in similar experimental conditions). Finally, drying time (or drying days), fresh mass, dry mass, water content, relative water loss rate (the ratio of the weight lost on the first day to the total fresh weight) and absolute speed of water loss of the fruits (water areal flux) were calculated.

Absolute water areal flux of ripe or near ripe fruits were measured using the formula

*V* = (*W*_1_ − *W*_2_)/*A* (1)

where *W*_1_ and *W*_2_ are the fresh weights at the start and after 1 day, respectively, and *V* and *A* are the absolute water loss rate and fruit surface area, respectively. Surface area *A* was estimated based on fruit shapes by measuring the length, width and height of the fruits. Due to the diversity and complexity of fruit shapes, surface area was calculated or estimated for regular shapes using the appropriate or standard geometric formulae (e.g., the surface area of a cone is given by the formula: *S* = π*rl* + π*r*^2^, where *r* is the radius of the base and *l* is the height; the surface area formula for an ellipsoid is given by the formula *S* ≈ 4π (*ab* + *bc* + *ca*)/3, where *a*, *b*, and *c* is three semi-axis length, respectively).

The determination of fruit maturity is mainly based on a series of comprehensive traits such as the changes in the fruit skin color (change more than 20% of fruit surface), fruit smell, and state of the naval and pedicel of fruits, combined with the phenological period [17,24], growth cycle, and pulp or seed state of other fruits on the same plant individual. Fruit maturity index (*M_f_*) was estimated based on following formula: *M_f_* _=_ *D_s_*/*D_w_* (*D_s_*: days from flower pollinated to sample time, *D_w_*: fruit development time). Based on these properties, fruit development stages such as at maturity (*M_t_* ranges from 0.8 to 1.0), before *M_t_* ranges from 0.4 to 0.8), or after fruit ripening, were determined.

### 2.4. Statistical Analysis

#### 2.4.1. Relationships Between Fruit Types and Shapes

All analyses were conducted in R (v. 4.3.0). We built a phylogenetic tree for all the species (29,760 in total) using the R package V.PhyloMaker2 (v. 0.1.0) [25]. Two traits were considered in our study: fruit types and fruit shapes. Both traits are categorical. For fruit types, we considered two categories: fleshy and dry. For fruit shapes, we considered four categories: globular or spheroidal (GS), elongated (E), flaky (F), and small globular (SG).

We conducted a measurement on phylogenetic signal (δ) developed by Borges et al. (2019) [26] using their R code available at https://github.com/mrborges23/delta_statistic (accessed on 23 October 2023). The value of δ is always greater than 0; the higher the value, the stronger the phylogenetic signal between a given tree and a trait. This measurement can be used for any categorical trait. For fruit types, which are binary traits, we also calculated another value (D) for phylogenetic signal, developed by Fritz and Purvis [27], using the R package caper (v.1.0.3) [28]. A trait with random association will have D ≈ 1, while a trait following the Brownian model will have D ≈ 0. Values of D smaller than 0 indicate that traits are more phylogenetically conserved than expected under the Brownian model, and values of D greater than 1 indicate traits that are phylogenetically over-dispersed.

We randomly selected 1000 species from the total 29,760 species and extracted a sub-tree to calculate the two values, δ and D. For each of these sub-trees we randomly shuffled the trait values and calculated a new value for δ. For D, we randomly selected 2000 species and also performed the same procedure. All calculations were repeated 30 times. Within each dataset, there are no repeated species. But between datasets, there may be some overlapping species.

To investigate the relationships between fruit types and fruit shapes, we built phylogenetic generalized linear (PGL) models (phylogenetically independent analysis) for the 29,760 species using the R package phylolm (v. 2.6.2) [29], which accounts for phylogenetic correlation between the species. In the model, fruit types (a binary variable with a value of 1 for fleshy fruits and 0 for dry fruits) were used as the dependent variable, examining the relationships between fruit types and fruit shapes.

#### 2.4.2. Fruits Drying Days and Their Responsible Factors

We also built a phylogenetic tree for the studied species using the R package V.PhyloMaker2 (v. 0.1.0) [25]. Before conducting the following analyses, we log-transformed all numeric variables. We tested the phylogenetic signal in drying time, fresh mass, dry mass, loss rate of fresh mass on the first day, proportion of dry mass, proportion of water, fruit length, fruit width, fruit height, fruit surface area, fruit size, fruit type and fruit shape using Pagel’s λ [30] and Blomberg’s K [31] calculated using the ‘phylosig’ function in the package ‘phytools’ v1.9-16 [32]. A λ or K of 0 indicates no phylogenetic signal [32]. For fruit types, we calculated the D values, and for fruit shapes, we calculated the δ vales, as described above.

We performed correlation analysis between all variables. For numeric variables, we used the Pearson correlation coefficient, implemented in the R package stats. We conducted association analysis between a binary (fruit types) or categorical variable (fruit shapes) and a numeric variable using mutual information, implemented in the R package mpmi (v. 0.43.2.1) [33].

To investigate the relationships between drying time and other variables, we built phylogenetic linear models (PLMs) using the R package phylolm (v. 2.6.2) [29], which incorporates phylogenetic correlations between species. Since significant correlations existed between almost all pairs of variables, we constructed 11 models with drying time as the dependent variable and one of the 11 variables, including fresh mass, dry mass, proportion of dry mass, proportion of water, fruit length, fruit width, fruit height, fruit surface area, fruit size (the sum of length, width, and height), fruit type, and fruit shape as independent variable.

To determine the variation explained by each independent variable in drying time, we used partial R^2^ values for the PLMs [34], implemented in the R package rr2 (v. 1.1.1) [35]. The partial R_lik_^2^ for each variable was calculated by comparing the full model (which is a PLM including the focal independent variable) with the reduced model in which the independent variable was removed and measuring the consequent reduction in likelihood. All models were constructed using the univariate framework described above. Similarly, we quantified the variation explained by phylogeny by comparing the full PLM, a reduced general linear model that included the corresponding independent variable but did not account for phylogenetic covariance. Additionally, PLMs were used to evaluate variation in the initial mass loss rate explained by fruit shape, type and surface area.

#### 2.4.3. Allometric Relationships Between Propagule Mass and Surface Area of Circular, Spherical and Other Forms of Fruits

Taking circular and spherical fruits as examples, when propagule mass increased by a factor of *n_i_* (*n_i_* > 1), surface areas of circular dry fruits would increase by *n_i_* times, while the surface areas of globular fleshy fruits would just increase by n_i_^2/3^ times (<*n_i_*). The details of the derivation are below.

For a circular fruit, when it grows up from initial weight (*W*_1_) to a certain weight (*W*_2_), its surface area (*S*_1_, *S*_2_), volumes (*V*_1_, *V*_2_) and weights are expressed as the following models:

*S*_1_ = π*r*_1_^2^, *S*_2_ = π*r*_2_^2^, *W*_1_ = *V*_1_ × *D*_1_ = *S*_1_ × *D*_1_, *W*_2_ = *V*_2_ × *D*_2_ = *S*_2_ × *D*_2_

where *r*_1_ and *r*_2_ are radii of the fruits in the two stages, *D*_1_ and *D*_2_ are densities of the fruits. If we assume *D*_1_ equals to *D*_2_, thickness is ignored (*S*_1_ = *V*_1_; *S*_2_ = *V*_2_), when *W*_2_ = *n*_i_ *W*_1_, then *S*_2_ = *n*_i_ × *S*_1_.

For a spherical fruit, when it grows up from initial weight (*W*_1_) to a certain weight (*W*_2_), its surface area (*S*_1_, *S*_2_), volumes (*V*_1_, *V*_2_) and weights are expressed as the following models:

*S*_1_ = 4π*r*_1_^2^, S_2_ = 4π*r*_2_^2^, *W*_1_ = *V*_1_ × *D*_1_ = 4/3 × π × *r*_1_^3^, *W*_2_ = *V*_2_ × *D*_2_ = 4/3 × π × *r*_2_^3^

where r_1_, r_2_, D_1_ and D_2_ are the same as the above. If we assume D_1_ equals to D_2_, when W_2_ = n_i_ W_1_, then S_2_ = n_i_^2/3^ S_1_.

Therefore, we suggested that the increase in surface area of fruits of other shapes is greater than that of spherical fruits as fruits develop.

## 3. Results

### 3.1. Water Content Shift Patterns of Fruits from Juvenile to Maturity as Well as After Maturity

**Figure 1:**
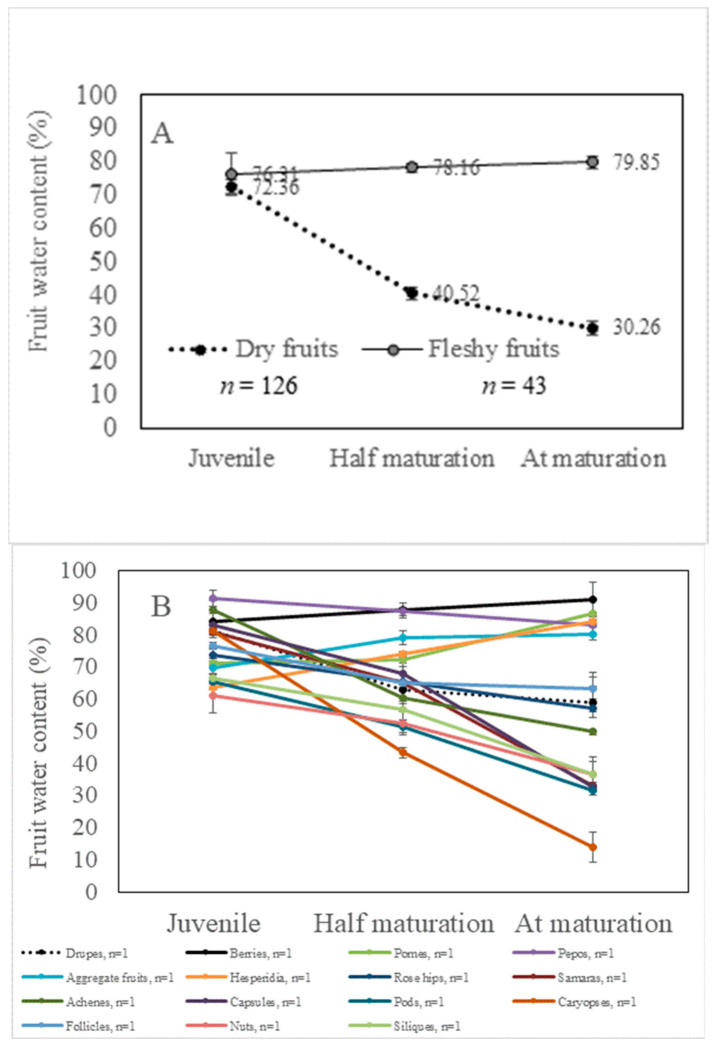
Water content shift trend chart of fleshy and dry fruits and various fruit types from juvenile (M_t_ ≤ 0.4) to half (0.4 < M_t_ < 0.8) and at maturation (0.8 ≤ M_t_ ≤ 1.0) (**A**) fleshy and dry drupes; (**B**) subtypes of fleshy and dry fruits. (The error bars are mean ± SD.).

Based on fruit water content throughout the fruit development process, water content of dry fruits decreases generally, while that of fleshy fruits (at least for pericarp of drupes and rosehips) increases slightly or remains constant with fruit maturation (Figure 1A). The majority of plant species with fleshy fruits i.e., (the sciophytes) with berries, pomes, pepos, hesperidium and aggregate fruits, exhibit little change in water content during development (Figure 1B). For example, fruit water content of *Lonicera fragrantissima* is 87.60 ± 1.92% before maturation and 91.04 ± 4.30% at maturation. Water content of drupes and rosehips decreases slightly with fruit maturation due to increase of fresh mass of seeds with lower water content, however, water content of the pericarp remains high from juvenile to maturation. For instance, fruit water content of *Prunus trilobata* is 81.30 ± 1.20% before maturation and 58.95 ± 4.83% at maturation. On the whole, fleshy fruits had a higher water content even at fruit maturity than the majority of dry fruits (Figure 1). However, water content of a small portion of dry fruits such as *Yulania denudate*, *Yulania biondii* (both with follicles) and *Gleditsia japonica* (with pods) remains high at maturity.

For dry fruits, water content before maturation is significantly higher than at maturity (*p* < 0.0001). For fleshy fruits, water content before maturity is significantly lower than at maturity (*p* = 0.0178). Before maturity, water content in fleshy fruits is significantly higher than that in dry fruits (*p* = 0.0076). At maturity, water content in fleshy fruits is also significantly higher than that in dry fruits (*p* < 0.0001). The decrease in water content from before maturity to at maturity for fleshy fruits is significantly lower than that for dry fruits (*p* < 0.0001) (Figure 1A and Figure S1).

**Figure 2:**
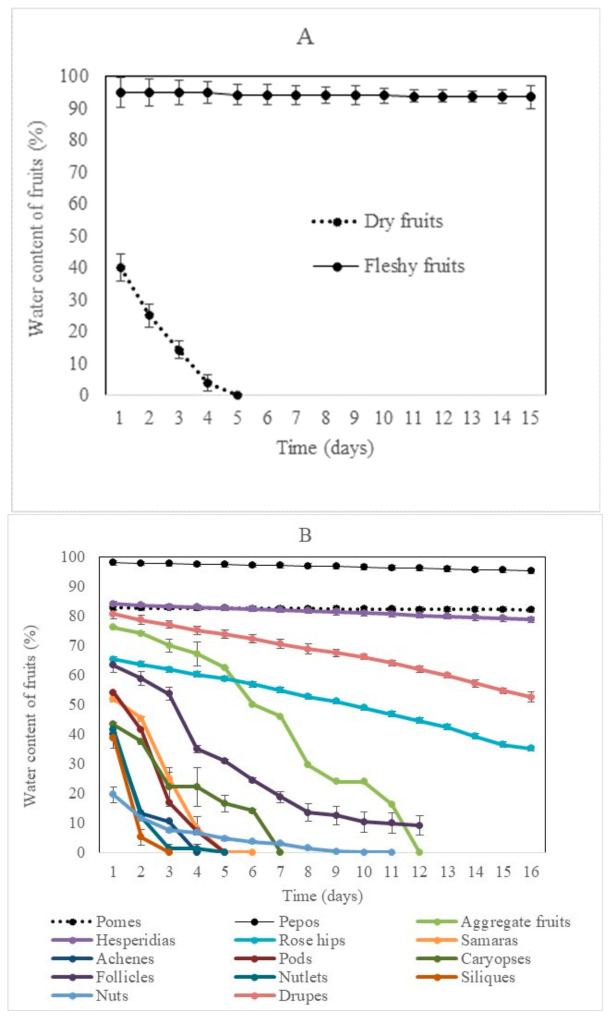
Water content shift with time for typical fleshy and dry fruits (such as berries and capsules) (**A**) and other fruit subtypes (**B**) after maturation (sketch map) (The error bars are mean ± SD).

Based on the speed of water loss from mature fruits, two general modes were found: rapid and slow (Figure 2A). Dry fruits exhibit rapid water loss, characterized by continuously declining water content from the highest value at juvenile to lowest value at maturity, followed by a rapid water loss after maturity; there is a sudden decrease of water content for some dehisced fruits. In contrast, fleshy mature fruits have slow rates of water loss or pericarp areal water flux (Figure 2A,B).

### 3.2. Fruit Drying Period and Correlations Between Relative and Absolute Speed of Fruit Water Loss

**Figure 3:**
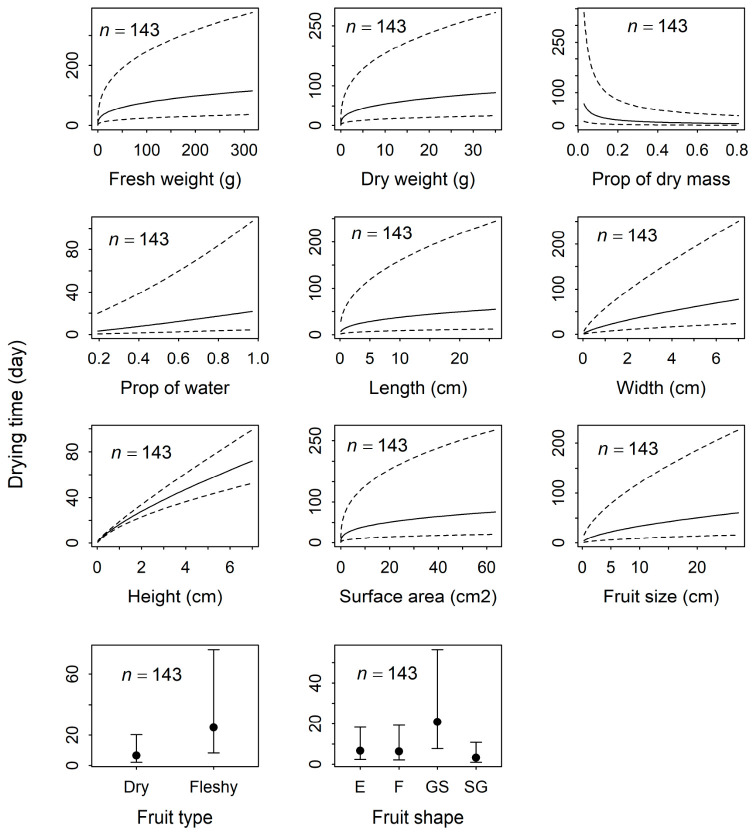
The response of fruit drying time to the independent variables: fresh weight, dry weight, proportion of dry mass, proportion of water, length, width, height, surface area, size, type and shape of fruits. Fruit shapes are classified as elongated (E), flaky (F), globular or spheroidal (GS) and small globular (SG). Solid lines and dots represent mean values and dashed lines and error bars (SD) indicate 95% confidence intervals.

Fruit water content ranged from 3.33% to 98.57% and the length of the drying period among the study species varied from 2 to 180 days. For instance, fruits of *Cocos nucifera* (a type of drupes), weighing over 3kg, were not dried after 6 months of air drying. Fleshy fruits and fruits with global shapes were found to have longer drying days than dry fruits and fruits with non-global shapes (Figure 3). Additionally, dry fruits of some species such as *Yulania denudate*, *Yulania biondii* and *Gleditsia japonica* have a longer fruit drying period than other dry fruits.

Significant correlations were found between length of fruit drying period and other fruit variables such as fresh mass, dry mass, proportion of dry mass, proportion of water, length, width, height, surface area and fruit size (Table 1, Figure 3, Figures S2 and S3). Significant phylogenetic signals were present in all variables (Table 2). Independent variables separately explained 7.9–48% of the variation in fruit drying time, while phylogeny explained 6–23% of the variation, except for fruit height.

**Figure 4:**
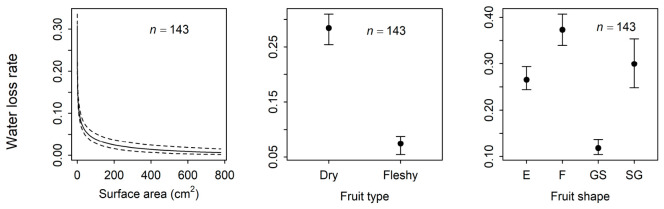
Response of relative water loss percentage to the independent variables such as fruit surface area, types and fruit shapes after one days. Fruit shapes are classified as elongated (E), flaky (F), globular or spheroidal (GS) and small globular (SG). Solid lines and dots represent mean values and dashed lines and error bars (SD) indicate 95% confidence intervals.

Fruit type, shape and surface area exhibited significant positive relationships with rate of fresh fruit decrease in mass on the first drying day (Figure 4 and Figure S4). In univariate frameworks, fruit type, shape and surface area explained 27.39%, 20.60% and 16.00% of the variation in mass loss, respectively. Significant phylogenetic signals were observed in all the variables, while the variation explained by the independent variables ranged from 21% to 49%. Variation explained by phylogeny remained relatively low, ranging from 3.8% to 4.4%.

**Figure 5:**
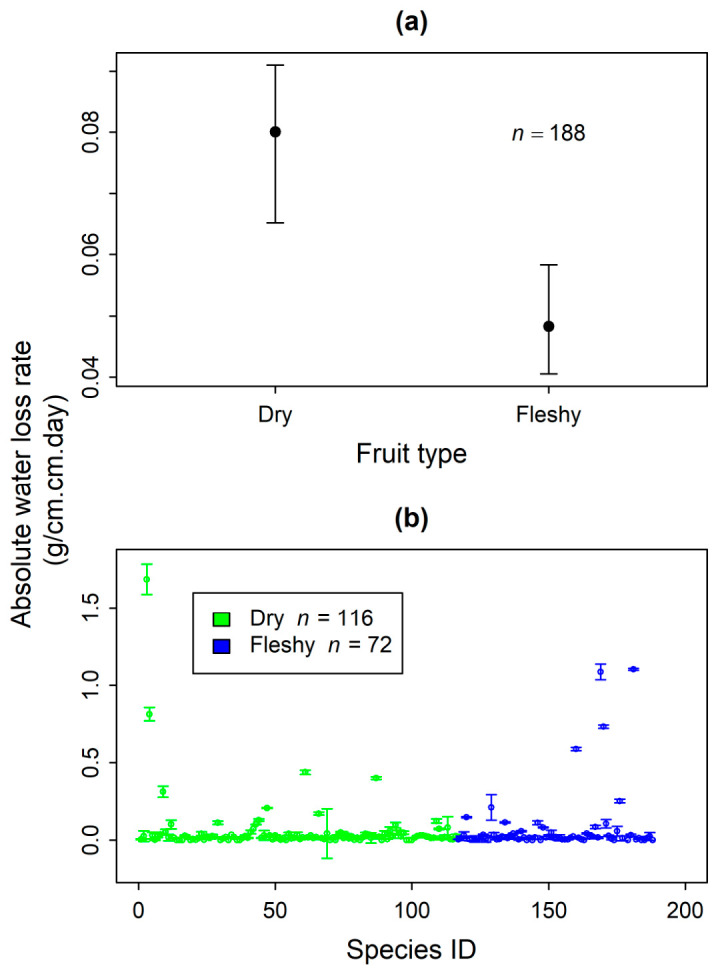
Absolute areal water flux through pericarp for dry and fleshy fruits: (**a**) significant difference between the two fruit types (*p* < 0.001, *n* = 188), (**b**) mean value (dots) with error bars showing standard deviations (mean ± SD) for each species (green, dry-fruited, *n* = 116; blue, fleshy-fruited, *n* = 72).

Dry fruits in the late stages of development have a significantly higher rate of absolute water loss than fleshy fruits (R = 2.15, *p* = 0.033) (Figure 5). The rate values of absolute water loss vary across different fruit developmental stages. Species with smooth and bright surfaces of fleshy fruits at fruit maturity (e.g., species in Caprifoliaceae, Solanaceae, Rosaceae, Cornaceae) have the lowest areal water flux through the pericarp. For dry fruits, the water flux of the pericarp decreases with time, whereas for fleshy fruits areal water flux through pericarp remains basically unchanged.

### 3.3. Phylogenetic Signal and Interaction of Fruit Types and Shapes

**Figure 6:**
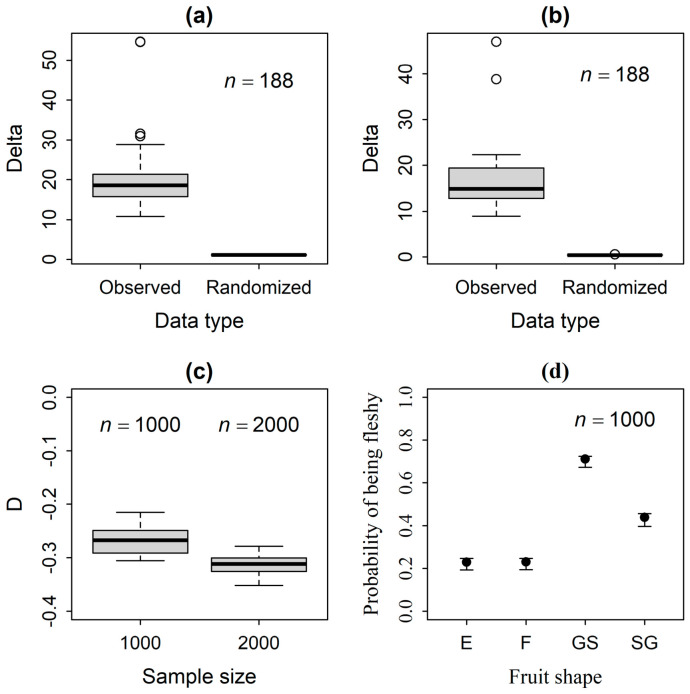
(**a**) Delta index measuring phylogenetic signal in fruit types. Thirty sets of 1000 species were randomly selected from the total pool of 29,760 species. For each set, the “observed” delta value was calculated and compared with delta value obtained after randomization of trait values. (**b**) Delta index measuring phylogenetic signal in fruit shapes, calculated using the same resampling and randomization procedure as in (**a**). (**c**) D index measuring phylogenetic signal in fruit types. Thirty sets of 1000 and 2000 species were randomly selected from the full data set and a D value was calculated for each set. (**d**) The probability (mean value and 95% confidence interval) that a species bears fleshy fruits fruit shapes are globular or spheroidal (GS), elongated (E), flaky (F), and small globular (SG).

For fruit types and fruit shapes, the phylogenetic signal delta values (δ) calculated with the resampled observed data are much larger than those calculated with the randomized data (Figure 6a,b and Figure S5). The D values are clearly smaller than 0 for both sample sizes (Figure 6c). These results demonstrate the presence of a phylogenetic signal in the two traits. Globular or spheroidal fruits show the highest probability of being fleshy, followed by small globular fruits, while elongated and flaky fruits show the lowest probability (Figure 6d and Figure S6). On the contrary, dry fruits tends to be elongated, flaky or small globular, and only a small fraction is globular.

## 4. Discussion

### 4.1. Water Content Shift Patterns of Dry and Fleshy Fruits and Mechanism During Fruit Development

Water content of dry fruits attached to the maternal plant decreased progressively from the juvenile stage to maturity under continuous water supply to the fruits (Figure 1A). In contrast, water content of fleshy fruit types (at least for pericarp of drupes etc.) increased slightly up to the point of ripeness, peaking at maturity or maintaining a consistent higher water content throughout development. These findings suggest distinct patterns of water content variation during fruit development for dry and fleshy fruits.

Pericarp areal water flux (or pericarp permeability) variation and fruit shape (essentially, the thickness of the pericarp) are considered as the two key factors involved in the patterns of water content shift (Figure 6d and Figure S2). For dry fruits with globular or small globular, increasing pericarp permeability due to intensified lignification with fruit development is the main responsible factor. For elongated and flaky dry fruits, water loss from the pericarp is enhanced due to allometric expansion between fruit surface area and podetium thickness or propagule mass. Based on Corner’s rule, stem/branch sizes should be positively related to their attached structures, such as leaves, flowers and propagules [15,36,37]. As an apparatus for mechanical support and transport water, podetium (a kind of twig) thickness (or cross-sectional areas of fruit pedicels) was positively related to propagule mass [19]. During fruit development, both the cross section of the podetium and fruit mass enlarge; however, surface areas of elongated or flaky dry fruits increase more than that of spherical or nearly globular fleshy fruits in the case of equal mass.

If we assume that fleshiness of fleshy fruits results from a water balance between transportation via xylem and phloem of the pedicels and loss via pericarp transpiration [38], then dryness of dry fruits can be attributed to water imbalance in water supply and loss. As dry fruits develop, the amount of water supplied by the fruit pedicels is less than that lost through the surface area of the fruits, leading to a gradual decrease in water content for dry fruits due to the accelerated expansion of the surface area of the non-spherical fruits.

The water content values (Ci, in %) of circular dry fruits can be expressed by the following equation to (see methods):

*C*_*i*_ ≈ *wn*_*i*_^−1/3^ (2)

where *w* is the water content (%) of the fruits during the initial young stage and *n*_i_ is the multiple by which the fruit mass increases (fruit thickness is ignored owing to small surface area produced by it). As the *n*_i_ value increases with fruit growth, water content *Ci* will decline. This may be one of the fundamental causes of decreasing water content in non-spherical dry fruit types. Our data from several fruit developmental stages observed for a few species (e.g., in Ulmaceae and Betulaceae) support the correctness of this model.

For dry fruits with various shapes (non-spherical), the above model may be transformed to the following equation:

*C*_*i*_ ≈ *wn*_*i*_^−m^ (3)

where m (0 < m < 1) is the shape coefficient of dry fruits with various shapes. Of course, the formula *C_i_* ≈ *wn_i_^−^*^m^ holds true when the pericarp water flux is consistent across developmental stages. However, the pericarp water flux varies across different stages of fruit development for dry fruits, and the above model may be transformed again to the following equation:

*C*_*i*_ ≈ *wp*_*i*_^−1^*n*_*i*_^−m^ (4)

where *p_i_ (p_i_* > 1, for dry fruits) is defined as the water variation coefficient of the pericarp related to areal water flux affected by fruit water content, fruit thickness and pericarp permeability, *p_i_* values will increase as dry fruits develop. For fleshy fruits, *p_i_* values are generally equal to or approximate to 1. For berries, drupes, pepos, pomes and hesperidiums, *p_i_* values can be less than 1 and their minimum values should be about 0.8 based on our observational data. This variation can help explain why the water content of globular dry fruits declines as they become mature.

In fact, the water inside fleshy fruits is not imbalanced. The increasing water content for the majority of fleshy fruit types (such as berries) indicates that input of water to the fruits is greater than output during fruit development due to lower pericarp water flux.

However, for plant species with drupes and rosehips, the increase or decrease pattern of the fruit water content depends on the proportion of seed mass relative to the total fruit mass. If the proportion of seed mass in fruits is sufficiently large, a decline pattern is followed. For instance, in *Prunus trilobata*, where the proportion exceeds 80%, water content declines as seed mass (which has lower water content at maturity, 56.70 ± 3.51%) becomes dominant. Additionally, the higher water content observed in the mature fruits of *Magnolia denudata* and *Magnolia biondii* (both with follicles, a kind of dry fruits) can be attributed to their higher fruit mass, lower pericarp areal water flux, and thicker pericarp.

Various fruit shapes display significant phylogenetic signals, serving as one of the key traits in the emergence of fleshiness and dryness for fleshy and dry fruits at maturity, indicating ancestral traits were derived from long-term adaptative evolution to heterogeneous habitats. In contrast to previous conclusions [16], we conclude that fleshy fruits, not dry fruits, are likely the primitive fruit types, since globular or spherical forms can evolve into flaky and elongated forms, whereas the revers transformation is almost impossible.

### 4.2. Classification of Fleshy and Dry Fruits and Its Significance

**Figure 7:**
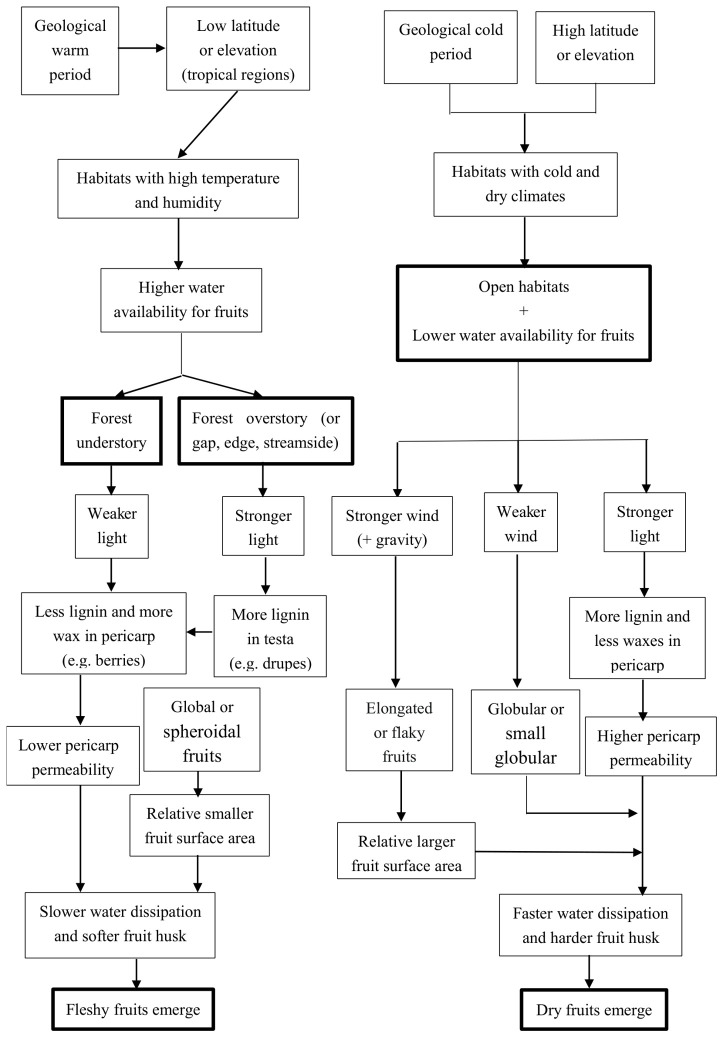
Biological and environmental mechanisms for development process of fleshy and dry fruits.

Our fruit drying experiment showed that mature fleshy fruits had a longer period of high-water content than dry fruits after the water supply was cut off. Several biotic factors may be responsible for this. First, the shapes of fleshy fruits tend to be spherical or nearly globular (Figure 6d), and globular spheres have the smallest surface area per unit mass. Second, we found that a higher fruit water content correlates with longer fruit freshness or fleshiness for both fruit types (Figure 3). In our study, water content of fleshy fruits (at least for pericarp for some fruit types, e.g., drupes, rosehips) reached a maximum when fruits were ripe, whereas that of dry fruits was very low at ripeness (Figure 1 and Figure 2). Third, duration of the fruit drying period is positively related to fruit height and mass, and fleshy fruits generally have greater height and mass than dry fruits (Figure S2) [5,9,19]. The thicker the fruits, the more difficult the loss of water through the pericarp, and the longer the fruit has fleshiness. Lastly, fleshy fruits often possess lower areal water flux through the pericarp than dry propagules in the later development or ripening stages (Figure 5) due to less lignin and more waxes or thicker cuticles in the pericarp of the fleshy fruits (Figure 7) [39].

Accordingly, fleshy fruits are suggested to be classified into three types based on the patterns of water content variation before maturity, their derivation habitats, and pericarp surface traits: (1) berries and aggregate fruits with waxed or membranous pericarp surface, likely originating in warm, humid and shaded habitats (forest understory), (2) pomes, pepos and hesperidium with leatherized pericarp, probably originating in warm, humid and half-open habitats, and (3) drupes and rosehips with lignified sporoderm, originating probably from warm and humid habitats with strong light (e.g., forest overstory), one of the direct evidences is that water content (or dry mass) of drupes and rosehips is significantly lower (higher) than that of berries (unpublished data) at maturity. Of course, further research is needed on the light conditions of the original habitat of drupes and rosehips. The evidence supporting this classification is found in two extant basal orders of terrestrial angiosperm: Amborellales (*Amborella trichopoda*) and Austrobaileyales (Austrobaileyaceae, Trimeniaceae and Schisandraceae). The former with a globular drupe is supposed to distributed in forest overstory and the later with a berry is supposed to distributed in forest middle layer (small trees) or forest understory (shrub, lianas or vines) [16,40]. Furthermore, dry fruits were suggested to be classified into two types according to water content at maturity and fruit drying period after maturity: (1) a small fraction of dry fruits such as follicles in Magnoliaceae and pods in Fabaceae (such as *Gleditsia sinensis* and *Styphnolobium japonicum*) with higher water content and fresh mass at maturity, originating possibly from warm and humid habitats with stronger light (e.g., forest overstory), and (2) other dry fruit types (such as capsules, achenes, samaras, pods, caryopses, nuts, cremocarps, follicles and utricles) with lower water content and fresh mass at maturity, originating possibly from colder, drier and open habitats. This classification of fleshy and dry fruits, unlike traditional methods based on morphological traits, can further advance our understanding on mechanisms in propagule size variation and fleshy-fruited distribution patterns.

Originating from open habitats [8], species with dry fruits have developed a series of strategies that facilitate propagule dispersal by wind, including elongated or flaky shapes, wing-like extensions or flanges, small fruit mass, low water content at fruit maturity, hard or fragile pericarp (in dehiscent fruits), and high pericarp water flux near or at fruit maturity even if when dry fruits have much lower water content than fleshy fruits (Figure 5). On the other hand, fleshy fruits attract birds and mammals due to their succulent pericarp, which has high water content and abundant nutrition (i.e., large propagule mass) [5,9,19]. In addition, the shape origin of fleshy or dry fruits controlled ultimately by genes [41] may be related to heterogeneous habitats respectively, due to role of drought [23] or wind (for instance, wind velocity, wind direction and their duration may affect the origin and evolution of fruit shapes, a concept we term the fruit shape origin hypothesis, which deserves further focus in the future).

### 4.3. Pericarp Areal Water Flux Variation of Fleshy and Dry Fruits and Their Environmental Mechanism

Our results showed that fleshy fruit pericarps displayed not only a low relative rate but also a low absolute rate of water loss in the later fruit development stages but prior to maturity. Although this is the result of the combined effects of several factors such as pericarp permeability, fruit water content, fruit thickness, as well as temperature, relative humidity and air flow speed of the surrounding environment, wherein pericarp permeability is supposed to be the most important among them. Low water loss indicates that the pericarp of fleshy fruits exhibits low permeability due to pericarp waxification or leatherization (less lignin content), which may be attributed to long term evolution to warm and humid (shaded or open) habitats or regions [42,43] (Figure 7). Moreover, paleontological evidence also suggests that early angiosperms fruits were adapted to high water availability and low evaporative demand [40] (Figure 7). Correspondingly, strong light in open habitats with cold and dry climates may lead to the evolution of higher pericarp permeability of dry fruits in the late stage of fruit development due to lignification occurring (being beneficial for water transport) in the pericarp [39,41] (Figure 7).

Moreover, low water loss indicates that the pericarp of fleshy fruits exhibits low permeability due to thicker cuticles or more wax content in the pericarps [43,44], which may have evolved due to higher air humidity and temperature on the surface of the fruits in warm and humid habitats rather than in cold and dry ones for a long time. The slow water loss of fleshy fruits initially suggests that the structure and amounts of waxes in the cuticle of the pericarps serve as a major barrier against water loss from fruits [45,46]. Variations in temperature among plant organs are related to their thickness, thermal capacity and configuration [47]. Therefore, the pericarp of globular or spherical dry fruits that originated in cold, dry and open habitats have more lignin and less wax, leading to higher areal water flux than the fleshy fruits evolved from warm and humid habitats. Additionally, water acts as an adhesive to some extent, helping to keep fruit bodies intact in shaded habitats. The declining water content for mature dry fruits may bring about a scarcity of water and wax (or other organic compounds) on the fruit surface, increasing their pericarp permeability or causing pericarp cracking [48].

Studies on angiosperm phylogeny have shown that some fleshy fruits (globular or spheroidal shapes, small globular shapes) arose in shaded habitats and disappeared in open habitats many times [8,16,20], suggesting that changes in lignin and wax content (or other wax traits) or cuticle thickness on the surface of fruits may play a key role due to habitat change or vegetation dynamics driven by climate [7] (see Figure 7). Consequently, their water availability modes would transform, for instance, dry-fruited species, like *Persicaria perfoliata* and *Carex baccans* (with nutlets), which have lived in shaded habitats for an extended period, may evolve into species with freshy fruits (e.g., drupes) [8,16] owing to lignin content reduction and wax content increment in the pericarp of fruits induced by shaded environment according to the punctuated equilibrium theory [49,50]. Nearly all mature dry fruit pericarps (such as the capsules of Liliaceae, Orchidaceae and Cucurbitaceae) of sciophytes were found to show a tendency toward waxification and fleshiness (i.e., higher water content). These results indicate that fruit types displaying significant phylogenetic signals (Figure 6a,c) are long-term evolutionary results that distribute in parallel habitats, respectively, favoring the species to form convergent traits [8,12]. This can be explained by key innovations (gene variation or mutation) induced by environmental pressure, for instance, the fleshy-fruited clade was found to contain more species than its dry-fruited sister clade in tropical understory plants [11].

## 5. Conclusions

Our findings provide important insight into the ecological mechanisms underlying the development process of fleshy and dry fruits, their repeated origins across taxonomic groups, and the evolutionary drivers of these fruit types in seed plants. Specifically, the distinct patterns of water content change during fruit development, variation in pericarp areal water flux (or pericarp permeability), and diversity of fruit shapes indicate that fleshiness of mature fruits is primarily determined by correlated evolution among plant organs in response to environmental adaptation, rather than by plant–animal interactions or seed dispersal alone. In detail, fruit type diversity is partly determined by fruit shape diversity and variation in pericarp areal water flux, both arising from allometric growth of lignified tissues in different plant organs across heterogeneous habitats. Development of dryness in dry fruits at maturity mainly results from declining fruit water content caused by progressive pericarp lignification and increased water dissipation during later developmental stages. This effect may be intensified by water imbalance between pedicel supply and surface-area-driven loss, especially in elongated or flaky fruits where pedicel diameter (or fruit mass) scales allometrically with fruit surface area. The imbalance also can be pronounced in globular or spheroidal fruits due to increased lignin accumulation and elevated pericarp water flux before maturation. Dry fruit development and evolution are often associated with cold, dry, and open habitats with strong light. In contrast, fleshy fruits develop through maintenance of a water balance between vascular supply from the pedicel or stem and reduced water loss through a waxy or leathery pericarp. This condition is commonly linked to globular or spherical shapes (i.e., greater thickness) and relatively low pericarp water flux. Fleshy fruits are generally considered as adaptations to warm and humid environments, either shaded habitats with weak light (e.g., sciophytes bearing berries, pomes, and pepos) or possibly open habitats with strong light such as forest canopies (e.g., heliophytes producing drupes and rosehips). Of course, much more evidence is required to support this last point.

There are several limitations in our study. First, patterns of water content change during fruit development—particularly in fleshy fruits—require further investigation, given the wide diversity of fruit types and limited number of species examined in this study. Second, three fruits per species is indeed a minimal sample size given the expected variation; however, we justified our sample size by comparing the data for interannual and intraspecific variations between different individuals for a few species, and not finding significant difference between them (confidence interval is 95%). Third, the relatively large temperature fluctuations caused by the non-strict artificial climate chamber conditions may have affected the accuracy of measured fruit water loss rates. However, the main conclusions remain robust given the substantial differences in water-related traits between fleshy and dry fruits at late developmental stages. Fourth, assessing fruit ripeness presents a challenge, particularly for dry fruits, but any potential bias in ripeness evaluation is unlikely to alter the fundamental conclusions of this study.

## Figures and Tables

**Table 1:** Pearson correlation coefficients for all pairwise combinations among fruit drying days and associated variables (fruit fresh weight, dry weight, prop. of dry mass, prop. of water, length, width, height, surface area and fruit size). All variables were log-transformed scale prior to analysis.

		Log Drying Days	Log Fresh Weight	Log Dry Weight	Log Proportion of Dry Weight	Log Proportion of Water	Log Length	Log Width	Log Height	Log Surface Area	Log Fruit Size
Log drying days	R		0.72	0.66	−0.35	0.19	0.37	0.60	0.74	0.55	0.53
	*p*		0	0	0	0.02	0	0	0	0	0
Log fresh weight	R	0.72		0.96	−0.32	0.14	0.64	0.71	0.76	0.77	0.75
	*p*	0		0	0	0.10	0	0	0	0	0
Log dry weight	R	0.66	0.96		−0.06	−0.09	0.64	0.69	0.74	0.76	0.74
	*p*	0	0		0.36	0.40	0	0	0	0	0
Log proportion of dry weight	R	−0.35	−0.32	−0.06		−0.84	−0.15	−0.19	−0.23	−0.19	−0.20
	*p*	0	0	0.46		0	0.07	0.02	0.01	0.02	0.02
Log proportion of water	R	0.19	0.14	−0.09	−0.84		0.06	0.04	0.05	0.06	0.07
	*p*	0.02	0.10	0.30	0		0.45	0.63	0.54	0.47	0.41
Log length	R	0.37	0.64	0.64	−0.15	0.00		0.53	0.43	0.89	0.94
	*p*	0	0	0	0.07	0.45		0	0	0	0
Log width	R	0.60	0.71	0.69	−0.19	0.04	0.53		0.81	0.86	0.74
	*p*	0	0	0	0.02	0.63	0		0	0	0
Log height	R	0.74	0.76	0.74	−0.23	0.05	0.43	0.81		0.70	0.66
	*p*	0	0	0	0.05	0.54	0	0		0	0
Log surface area	R	0.55	0.77	0.76	−0.19	0.06	0.89	0.86	0.70		0.96
	*p*	0	0	0	0.02	0.47	0	0	0		0
Log fruit size	R	0.53	0.75	0.74	−0.20	0.07	0.94	0.74	0.66	0.96	
	*p*	0	0	0	0.02	0.41	0	0	0	0	

**Table 2:** Mutual information (MI) between fruit types and each of the other numeric variables (fruit drying days, fresh weight, dry weight, proportion of dry mass, proportion of water, length, width, height, surface area, and fruit size). All numeric variables were log-transformed before analysis.

		Log Drying Days	Log Fresh Weight	Log Dry Weight	Log Proportion of Dry Weight	Log Proportion of Water	Log Length	Log Width	Log Height	Log Surface Area	Log Fruit Size
Fruit types	MI	0.26	0.07	0.04	0.10	0.08	0.05	0.08	1.207775 × 10^−10.16^	0.05	0.05
	*p*-value	0.00	0.12	0.22	0.00	0.01	0.10	0.01	0.00	0.07	0.06
Fruit shapes	MI	0.36	0.18	0.16	0.10	0.10	0.17	0.17	0.26	0.15	0.13
	*p*-value	0.00	0.01	0.01	0.11	0.05	0.00	0.002	3.353756 × 10^−6^	0.01	0.01

## Data Availability

The data for this article were uploaded in the Dataset of Dryad and are available on the following website: (DOI: 10.5061/dryad.qjq2bvqvs, 2025-08-04 data).

## Supplementary Materials

The following supporting information can be downloaded at https://www.mdpi.com/article/10.3390/plants15142216/s1, Figure S1: Water content of fleshy and dry fruits from juvenile (BM) to maturation (AM); Figure S2: Scatter plots for all pairwise combinations among fruit variables, including drying days (DD), fresh weight (FWt), dry weight (DWt), proportion of dry mass (PDMs), proportion of water (PW), length (Len), width (Wd), height (Ht), surface area (SA), and fruit size (Sz). All variables are shown on the log-transformed scale; Figure S3: Fresh and dry weight and height (thickness) of fleshy and dry fruits at maturity (mean values and 95% confidence inter-vals); Figure S4: Response of water loss rate to the independent variables: initial fresh weight, fruit types and fruit shapes (mean values and 95% confidence intervals); Figure S5: (a) Delta index measuring phylogenetic signal in growth form. Thirty sets of 1000 species were randomly selected from the total pool of 29,760 species. For each set, the “observed” delta value was calculated and compared with a delta value obtained after randomization of trait values. (b) The probability (mean value and 95% confidence intervals) that a species bears fleshy fruits across different growth forms: tree (T), shrub (S), herb (H), and climber (C); Figure S6: Variation in fruit drying, mass, water content, and size-related traits among fruit types (FT) and fruit shape categories (SP). Boxplots are shown for drying days (DD), fresh weight (FWt), dry weight (DWt), proportion of dry mass (PDMs), proportion of water (PW), length (Len), width (Wd), height (Ht), surface area (SA), and fruit size (Sz). Fruit types comprise dry (d) and fleshy (f) categories and are displayed on the x-axis. Within each fruit type, separate boxplots represent elongated (E), flaky (F), globular or spheroidal (GS), and small globular (SG) fruit shapes. Boxes represent the 25th–75th percentiles, centre lines indicate medians, whiskers extend to 1.5 × the interquartile range, and points denote outliers; Figure S7: Variation in fruit drying, mass, water content, and size-related traits among fruit shape categories (SP) and fruit types (FT). Boxplots are shown for drying days (DD), fresh weight (FWt), dry weight (DWt), proportion of dry mass (PDMs), proportion of water (PW), length (Len), width (Wd), height (Ht), surface area (SA), and fruit size (Sz). Fruit shapes comprise elongated (E), flaky (F), globular or spheroidal (GS), and small globular (SG) categories and are displayed on the x-axis. Within each shape category, dry (d) and fleshy (f) fruit types are shown separately. Boxes represent the 25th–75th percentiles, centre lines indicate medians, whiskers extend to 1.5 × the interquartile range, and points denote outliers; Table S1: Pearson correlation coefficients of fruit drying days with variables (fruit fresh weight, dry weight, proportion of dry weight, proportion of water, length, width, height, surface area and fruit size) in the original scale.
