# Supplementary material for: Water Content Variation Patterns of Fruits During Development and Their Effects on Fleshiness or Dryness for Fruits at Maturity
Source: Plants (Basel). 2026 Jul 21;15(14):2216. doi: 10.3390/plants15142216 (PMC13417427; doi:10.3390/plants15142216)
Supplement: Supplementary file 1 [file plants-15-02216-s001.zip › plants-4130458-supplementary.pdf]

# Supplements

**Table S1.** Pearson correlation coefficients of fruit drying days with variables (fruit fresh weight, dry weight, proportion of dry weight, proportion of water, length, width, height, surface area and fruit size) in the original scale.

| | | Drying days | Fresh weight | Dry weight | Proportion of dry weight | Propor-tion of water | Length | Width | Height | Surface area | fruit size |
|---|---|---|---|---|---|---|---|---|---|---|---|
| Drying days | R | 1.00 | 0.42 | -0.39 | 0.30 | 0. | 0.33 | 0.67 | 0.72 | 0.55 | 0.58 |
| | *p* | NA | 0.00 | 0.00 | 0.00 | 0.00 | 0.00 | 0.00 | 0.00 | 0.00 | 0.00 |
| Fresh weight | R | 0.42 | 1.00 | -0.95 | 0.09 | 0.09 | 0.35 | 0.77 | 0.78 | 0.91 | 0.63 |
| | *p* | 0.00 | NA | 0.00 | 0.01 | 0.01 | 0.00 | 0.00 | 0.00 | 0.00 | 0.00 |
| Dry weight | R | 0.39 | 0.95 | 1.00 | 0.03 | 0.03 | 0.28 | 0.71 | 0.71 | 0.85 | 0.55 |
| | *p* | 0.00 | 0.00 | NA | 0.52 | 0.54 | 0.00 | 0.00 | 0.00 | 0.00 | 0.00 |
| Proportion of dry weight | R | -0.30 | -0.09 | -0.03 | 1.00 | -0.99 | -0.09 | -0.14 | -0.16 | -0.12 | -0.14 |
| | *p* | 0.00 | 0.00 | 0.05 | NA | 0.00 | 0.60 | 0.10 | 0.06 | 0.17 | 0.22 |
| Proportion of water | R | 0.31 | 0.09 | 0.03 | -0.99 | 1.00 | 0.07 | 0.14 | 0.16 | 0.12 | 0.12 |
| | *p* | 0.00 | 0.00 | 0.54 | 0.00 | NA | 0.59 | 0.13 | 0.06 | 0.17 | 0.22 |
| Length | R | 0.33 | 0.35 | 0.28 | -0.09 | 0.07 | 1.00 | 0.46 | 0.42 | 0.61 | 0.89 |
| | *p* | 0.00 | 0.00 | 0.00 | 0.64 | 0.59 | NA | 0.00 | 0.00 | 0.00 | 0.00 |
| Width | R | 0.67 | 0.77 | 0.71 | -0.14 | 0.14 | 0.46 | 1.00 | 0.96 | 0.89 | 0.81 |
| | *p* | 0.00 | 0.00 | 0.00 | 0.13 | 0.013 | 0.00 | NA | 0.00 | 0.00 | 0.00 |
| Height | R | 0.72 | 0.78 | 0.71 | -0.16 | 0.16 | 0.42 | 0.96 | 1.00 | 0.85 | 0.78 |
| | *p* | 0.00 | 0.00 | 0.00 | 0.06 | 0.06 | 0.00 | 0.00 | NA | 0.00 | 0.00 |
| Surface area | R | 0.55 | 0.91 | 0.85 | -0.12 | 0.12 | 0.61 | 0.89 | 0.85 | 1.00 | 0.85 |
| | *p* | 0.00 | 0.00 | 0.00 | 0.17 | 0.17 | 0.00 | 0.00 | 0.00 | NA | 0.00 |
| Fruit size | R | 0.58 | 0.63 | 0.55 | -0.14 | 0.12 | 0.89 | 0.81 | 0.78 | 0.85 | 1.00 |
| | *p* | 0.00 | 0.00 | 0.00 | 0.22 | 0.21 | 0.00 | 0.00 | 0.00 | 0.00 | NA |

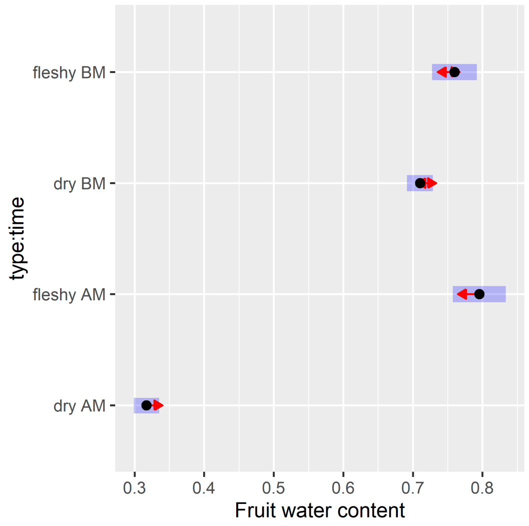


**Figure S1.** Water content of fleshy and dry fruits from juvenile (BM) to maturation (AM).

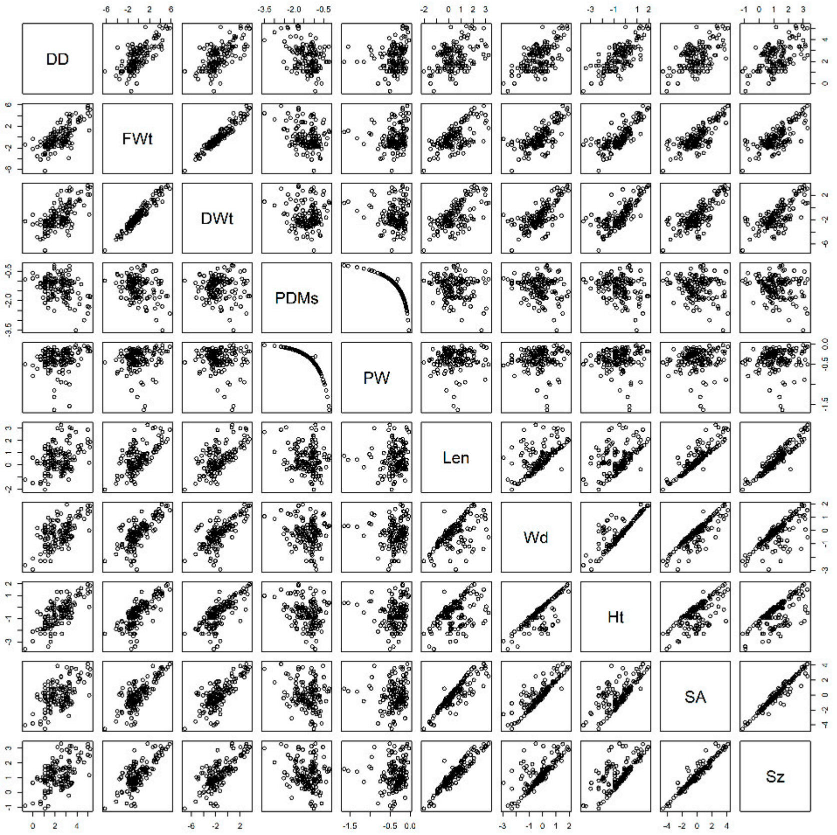


**Figure S2.** Scatter plots for all pairwise combinations among fruit variables, including drying days (DD), fresh weight (FWt), dry weight (DWt), proportion of dry mass (PDMs), proportion of water (PW), length (Len), width (Wd), height (Ht), surface area (SA), and fruit size (Sz). All variables are shown on the log-transformed scale.

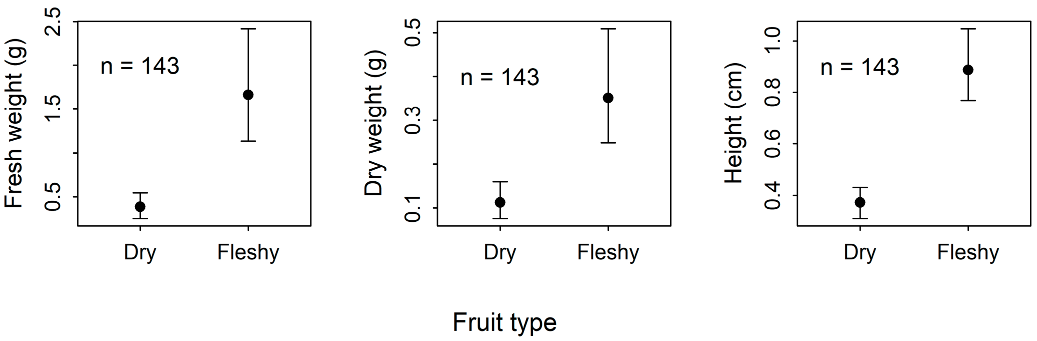


**Figure S3.** Fresh and dry weight and height (thickness) of fleshy and dry fruits at maturity (mean values and 95% confidence intervals).

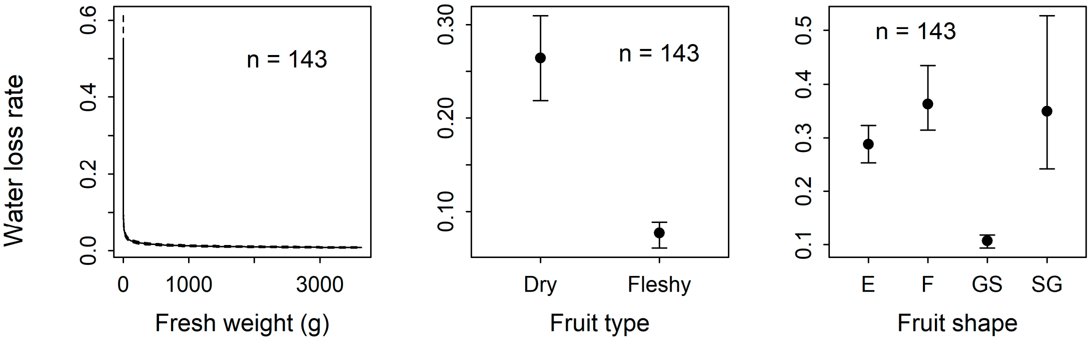


**Figure S4.** Response of water loss rate to the independent variables: initial fresh weight, fruit types and fruit shapes (mean values and 95% confidence intervals).

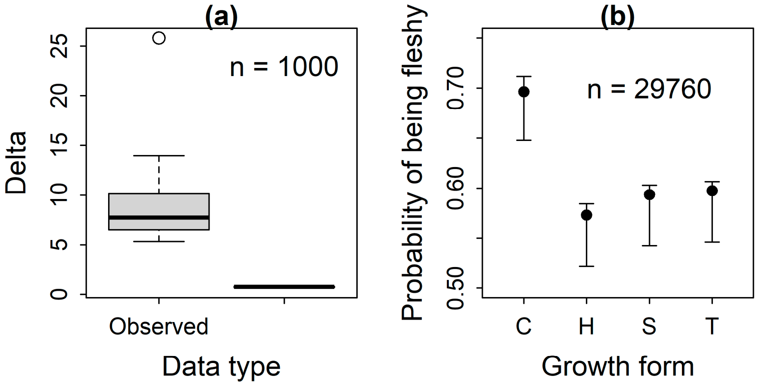


**Figure S5.** (**a**) Delta index measuring phylogenetic signal in growth form. Thirty sets of 1000 species were randomly selected from the total pool of 29,760 species. For each set, the "observed" delta value was calculated and compared with a delta value obtained after randomization of trait values. (**b**) The probability (mean value and 95% confidence intervals) that a species bears fleshy fruits across different growth forms: tree (T), shrub (S), herb (H), and climber (C).

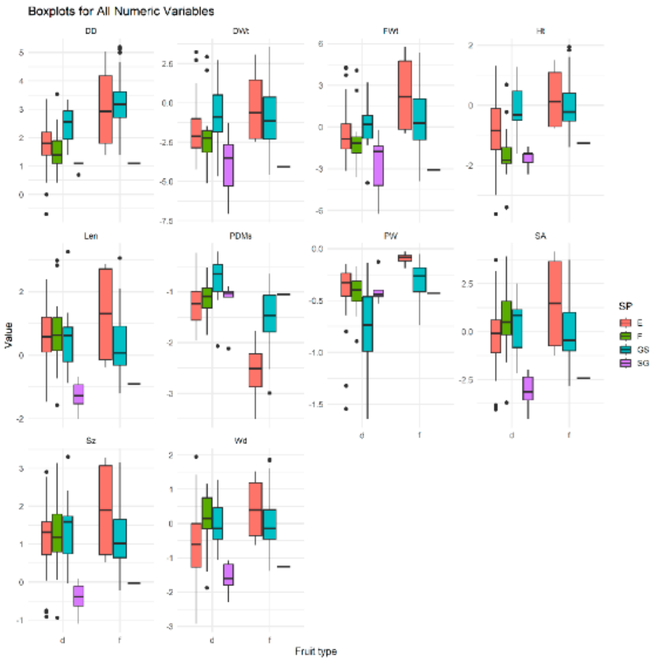


**Figure S6.** Variation in fruit drying, mass, water content, and size-related traits among fruit types (FT) and fruit shape categories (SP). Boxplots are shown for drying days (DD), fresh weight (FWt), dry weight (DWt), proportion of dry mass (PDMs), proportion of water (PW), length (Len), width (Wd), height (Ht), surface area (SA), and fruit size (Sz). Fruit types comprise dry (d) and fleshy (f) categories and are displayed on the x-axis. Within each fruit type, separate boxplots represent elongated (E), flaky (F), globular or spheroidal (GS), and small globular (SG) fruit shapes. Boxes represent the 25th–75th percentiles, centre lines indicate medians, whiskers extend to 1.5 × the interquartile range, and points denote outliers.

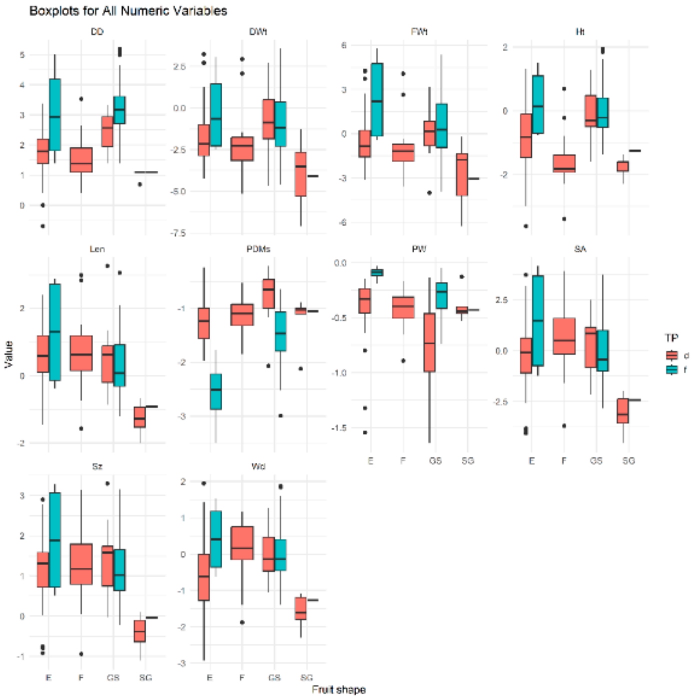


**Figure S7.** Variation in fruit drying, mass, water content, and size-related traits among fruit shape categories (SP) and fruit types (FT). Boxplots are shown for drying days (DD), fresh weight (FWt), dry weight (DWt), proportion of dry mass (PDMs), proportion of water (PW), length (Len), width (Wd), height (Ht), surface area (SA), and fruit size (Sz). Fruit shapes comprise elongated (E), flaky (F), globular or spheroidal (GS), and small globular (SG) categories and are displayed on the x-axis. Within each shape category, dry (d) and fleshy (f) fruit types are shown separately. Boxes represent the 25th–75th percentiles, centre lines indicate medians, whiskers extend to 1.5 × the interquartile range, and points denote outliers.
